# A Worker-Centered Personal Health Record App for Workplace Health Promotion Using National Health Care Data Sets: Design and Development Study

**DOI:** 10.2196/29184

**Published:** 2021-08-04

**Authors:** Hyun Sang Park, Kwang Il Kim, Ho-Young Chung, Sungmoon Jeong, Jae Young Soh, Young Ho Hyun, Hwa Sun Kim

**Affiliations:** 1 Digital Healthcare Department BIT Computer Co. Ltd. Seoul Republic of Korea; 2 Department of Medical Informatics Kyungpook National University Daegu Republic of Korea; 3 Finance Programs Department Korea Occupational Safety and Health Agency Ulsan Republic of Korea; 4 Elecmarvels Co. Ltd. Daegu Republic of Korea

**Keywords:** personal health record app, workplace health promotion, Fast Healthcare Interoperability Resources, national health care data set, human-centered design

## Abstract

**Background:**

Personal health record (PHR) technology can be used to support workplace health promotion, and prevent social and economic losses related to workers’ health management. PHR services can not only ensure interoperability, security, privacy, and data quality, but also consider the user’s perspective in their design.

**Objective:**

Using Fast Healthcare Interoperability Resources (FHIR) and national health care data sets, this study aimed to design and develop an app for providing worker-centered, interconnected PHR services.

**Methods:**

This study considered the user’s perspective, using the human-centered design (HCD) methodology, to develop a PHR app suitable for occupational health. We developed a prototype after analyzing quantitative and qualitative data collected from workers and a health care professional group, after which we performed a usability evaluation. We structured workers’ PHR items based on the analyzed data, and ensured structural and semantic interoperability using FHIR, Systematized Nomenclature of Medicine–Clinical Terms (SNOMED-CT), and Logical Observation Identifiers Names and Codes (LOINC). This study integrated workers’ health information scattered across different Korean institutions through an interface method, and workers’ PHRs were managed through a cloud server, using Azure API for FHIR.

**Results:**

In total, 562 workers from industrial parks participated in the quantitative study. The preferred data items for PHR were medication, number of steps walked, diet, blood pressure, weight, and blood glucose. The preferred features were ability to access medical checkup results, health information content provision, consultation record inquiry, and teleconsultation. The worker-centered PHR app collected data on, among others, life logs, vital signs, and medical checkup results; offered health care services such as reservation and teleconsultation; and provided occupational safety and health information through material safety data sheet search and health questionnaires. The app reflected improvements in user convenience and app usability proposed by 19 participants (7 health care professionals and 12 end users) in the usability evaluation. The After-Scenario Questionnaire (ASQ) was evaluated with a mean score of 5.90 (SD 0.34) out of 7, and the System Usability Scale (SUS) was evaluated a mean score of 88.7 (SD 4.83) out of 100.

**Conclusions:**

The worker-centered PHR app integrates workers’ health information from different institutions and provides a variety of health care services from linked institutions through workers’ shared PHR. This app is expected to increase workers’ autonomy over their health information and support medical personnel’s decision making regarding workers’ health in the workplace. Particularly, the app will provide solutions for current major PHR challenges, and its design, which considers the user’s perspective, satisfies the prerequisites for its utilization in occupational health.

## Introduction

### Background

Changes in lifestyle habits and the spread of chronic diseases have increased health problems within companies [1]. Workforce health is increasingly important for market relevance; the World Health Organization (WHO) showed the physical and mental health of workers to be imperative to companies’ success and competitive edge [2]. Compared with the general public, workers are at an increased risk of stress caused by a heavy workload and unhealthy lifestyle, including lack of exercise and frequent drinking [3]. Workers’ health may be directly or indirectly linked to work efficiency, corporate productivity, and industrial accidents beyond the individual level. Managing workers’ health at the corporate level can prevent social and economic losses, and employers are increasingly interested in improving workers’ health and welfare as a corporate strategy [4-6].

The workplace, where workers spend most time [7], is the best place to apply the concept of health promotion. The concept of workplace health promotion denotes that employers, workers, and local communities work together to improve workers’ mental and physical health and welfare [8]. Workplace health promotion initiatives can foster an appropriate work environment and promote personal health management [9,10]. Its primary challenge is increasing worker participation; studies have shown participation rates of less than 50% [11] and average annual reduction rates of 28% [12]. These obstacles can be overcome by applying health care technology to workplace health promotion [13].

Applications of health care technology, such as the personal health record (PHR), can increase workers’ interest, motivation, and participation in workplace health promotion [14,15] through its technology-based attributes [16]. PHR allows users to systematically collect, process, store, and share their health information with others, such as family members or medical personnel [17]. PHR users can easily access their medical records, prescription drug information, hospital test results, and health promotion information [18]. Given that the use of PHR promotes cooperation between medical personnel and workers through communication, it can help reduce medical expenses and strengthen disease prevention, management, and treatment activities [19,20]. Because of the expected effects of PHR, it is increasingly provided by employers as part of self-managed health care programs [21,22].

PHR is intended to help workers manage their health information, but privacy concerns have evoked obstacles to its use [21]. Workers are often reluctant to allow employers to access their PHR, raising direct practical problems [23]. Concerns about the exposure of personal information and fear of discrimination are often discussed as privacy and security issues of PHR [24], and workers may question the motives of employers who provide such services [25]. Various factors influence PHR system acceptance and use [21], with workers’ acceptance of PHR being influenced by individual and organizational factors (eg, trust in employer, management support for PHR, communication, and awareness), along with technical factors [16]. Workers’ participation depends on incentive provision and how PHR is presented to them [26].

Privacy issues, lack of motivation, and operational difficulties have been identified as major obstacles for the use of PHR [27], with various studies promoting the use of PHR. Pushpangadan and Seckman [28] argued that consumer adoption was slow because PHR was designed based on a clinically oriented design, without considering the consumers’ perspective. Weinert and Cudney [29] showed that PHR efficiency depends not only on system evolution and complexity, but also on user-friendliness, easy-to-use design, and structured documents. Thus, developing a successful PHR app may entail considering users’ perspectives from the design stage, coupled with a systematic design methodology.

In terms of data access, workers currently must collect their health information from individual institutions and workplaces, which complicates individuals’ active participation in their health management. Interconnected PHR services, where workers collect and manage their health information in one place, with users controlling others’ access to their information, may provide a solution to this challenge. Data exchange based on workers’ authorization is possible only when the structural and semantic interoperability of the PHR is guaranteed. Interoperability [30-32] is important in workers’ adoption of PHR and is known to be a major challenge for PHR, in addition to security and privacy [33] and data quality [17]. A successful workplace PHR app service can be developed and operated by making the app user centric, and ensuring interoperability, security and privacy, and data quality.

This study aimed to design and develop a PHR app providing a worker-centered interconnected PHR service. To this end, we designed a PHR app following the analysis of quantitative and qualitative data collected from workers and a group of health care professionals, employing the human-centered design (HCD) methodology. We developed the app based on national health care data sets using web technologies.

### Prior Work

Studies have been conducted to standardize PHR and address interoperability issues. Simon et al [34] developed a PHR that acquires measured data from a device through IEEE 11073, converts them to ASTM continuity of care record (CCR), and transmits them to a server. Marceglia et al [35] proposed a design based on Health Level Seven (HL7) clinical document architecture (CDA) that can be adopted when exchanging information between PHR and electronic health records (EHRs). Plastiras and O’Sullivan [36] developed an ontology-based architecture model that can ensure interoperability between PHR and EHR using various standards, such as CCR and CDA. Li [37] proposed a mobile PHR using various standards such as CDA, Digital Imaging and Communications in Medicine (DICOM), Systematized Nomenclature of Medicine–Clinical Terms (SNOMED-CT), and Logical Observation Identifiers Names and Codes (LOINC).

Since the introduction of Fast Healthcare Interoperability Resources (FHIR), studies have been conducted to apply it to PHR. Hong et al [38] developed a PHR system using FHIR and internet of things cloud to build an interconnected PHR, thereafter conducting a clinical trial to develop an obesity management model for 500 patients. Saripalle et al [39] developed a prototype of a tethered mobile PHR using FHIR and OpenEMR, and the developed mobile PHR synchronizes user data stored in OpenEMR using an HL7 Application Programming Interface (HAPI) library [40].

Studies have been conducted to develop PHR by applying various design approaches. Farinango et al [41] developed a PHR system for metabolic syndrome management by applying the HCD methodology. Farinango et al further developed 3 prototypes through 5 iterations by collecting user information through a survey of 1187 respondents, 8 interviews, and focus group interviews (FGIs) with 7 people. Zhou et al [42] developed and evaluated a mobile PHR app through a user-centered design (UCD) methodology, which involved using survey data from 609 respondents, and then conducting a usability evaluation on 15 participants. The UCD methodology has been used in other studies as well. For instance, Massoudi et al [43] developed a PHR that supports lifestyle intervention by applying the UCD methodology. They conducted structured interviews with 42 participants (28 users, 8 health care professionals, and 6 personal trainers) and user tests on 16 participants. Marchak et al [44] also applied the UCD methodology to develop and evaluate a web-based PHR for survivors of childhood cancer; they conducted FGIs and structured interviews with 28 patients (3 patients with pediatric cancer, 11 parents, and 14 health care providers), and a usability evaluation with 16 participants.

Various studies have also been conducted on workers and employers to operationalize the PHR. Dawson et al [22] conducted a questionnaire survey to understand workers’ perceptions (in large companies) of PHR; results showed that the reason for the low confidence in the PHR was a lack of trust in employers and other employees who may have access to employees’ health information. Fernando et al [45] analyzed the demographic characteristics of workers and health-related productivity (absence and overwork) related to PHR; results showed that high performers had a high absenteeism rate, indicating that PHR needs to focus on high performers. Fernando et al [46] also conducted quantitative and qualitative research on workers and employers to design the data model of PHR, thereafter developing and evaluating web-based PHR prototypes [47].

### Fast Healthcare Interoperability Resources

Occupational factors, such as patients’ workplace environment, need to be considered when managing chronic diseases; thus, occupational information has been integrated into the EHR [48] or an occupational data for health model [49]. HL7 has been used to design an FHIR profile [50] to represent patients’ occupational elements in PHR. The FHIR [51] was developed by HL7 in 2014 and is a next-generation standard for EHR exchange. It utilizes a reference information model, lightweight web services, and the latest web and app development principles. It was developed based on lessons learned from the HL7 standard and expert experiences. V2, which focused on the message-based exchange, required customization owing to semantic inconsistencies in its implementation [52]. The V3 reference information model provided a framework for expressing semantically consistent clinical statements, but owing to the complexity of its implementation, compatibility between system and document was hindered [53,54]. The FHIR was designed to be concise and easy to understand by adopting the advantages of the existing HL7 standard.

The FHIR simplifies various types of information generated in the medical field and expresses all contents as exchangeable resources. Each resource has its original form, and they refer to the URL of a resource only when the content of another resource is needed. When FHIR expresses EHR, it is expressed as a combination of various resources, such as Lego blocks, so that information can be easily recycled and only the necessary resources can be updated. Currently, there are over 150 resources, including clinical concepts (eg, allergy, condition, family member history, medication, and observation) and administrative information (eg, patient, practitioner, organization, and location). These resources are provided to external systems and clients through RESTful application programming interfaces (APIs). Regarding data exchange, transport layer security should be used, with OpenID Connect and OAuth being recommended for user identification, authentication, and authorization.

## Methods

### Service Design

**Figure 1:**
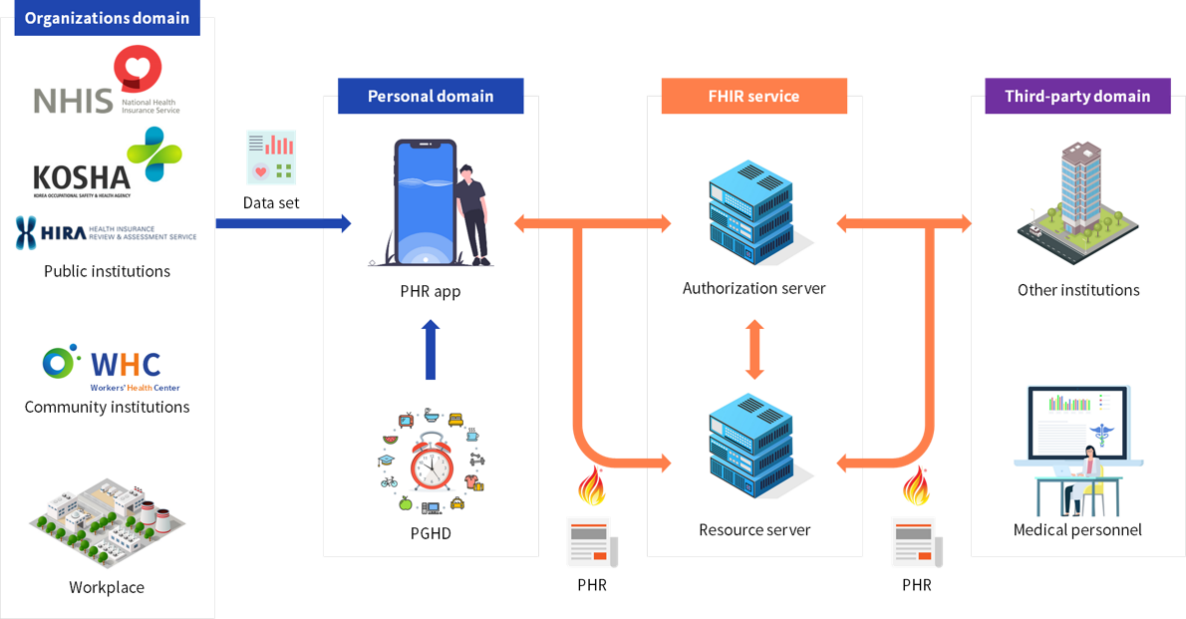
Conceptual diagram of a worker-centered personal health record service. FHIR: Fast Healthcare Interoperability Resources; PGHD: patient-generated health data; PHR: personal health record.

Worker-centered PHR services ensure continuity of care outside the workplace by allowing workers to easily collect their health information from various sources and manage it as PHR (Figure 1). Although PHR has become technically safe, users still must manually input their data [38]. Generally, health information is generated from a variety of sources (eg, health care providers, insurance companies, social networks, mass media, and public institutions) [55], and the generation of interoperable PHR requires the integration of data from different sources [56].

This study collected users’ health information through an API, and used an authentication method set by each institution. Institutions were classified into public and community institutions and workplaces, according to the data management entity. Public PHR sources were the National Health Insurance Service, Korea Occupational Safety and Health Agency, and the Health Insurance Review and Assessment Service. These institutions manage medical treatment history, prescription history, medical checkup results, and medical institution information according to the role of the institution, and users can view the data at their request. These data are national data generated when persons eligible for national health insurance access services provided by medical institutions.

Workers’ health centers are community institutions in Korea that provide services to prevent occupational diseases among workers in industrial parks (incorporating various industries, including manufacturing plants and factories). Currently, there are 23 centers in operation. Each institution comprises professional personnel, such as occupational and environmental medicine specialists, occupational nurses, industrial hygiene safety engineers, physical therapists, and counseling psychologists, who provide comprehensive occupational health services, including occupational, cerebrovascular, and musculoskeletal disease prevention, and job stress prevention. All workers can visit their nearest center and use its services free of charge, similar to a workplace infirmary. Workers’ health centers systematically manage the information of workers and workplaces in their area through an integrated system [57].

The workplace refers to the company employing the worker, where an occupational health manager manages workers’ information generated through the workplace health promotion program. As such, workers’ information is scattered across various sources, and needs to be managed in an integrated manner to ensure effective workplace health promotion.

Unlike an EHR, PHR can add patient-generated health data (PGHD). The PHR app from this study acquired data using various devices (eg, smartphone sensors, wearable bands, blood pressure monitors, blood glucose meters, and scales) and integrated these data with health information collected from each institution. These integrated data can be converted into an FHIR-compliant PHR according to the users’ needs, and then managed through a cloud service. The FHIR service comprises authentication procedures and resource servers that allow safe data management in the cloud by restricting access to users’ resources only to authorized institutions.

### Design Methodology

**Figure 2:**
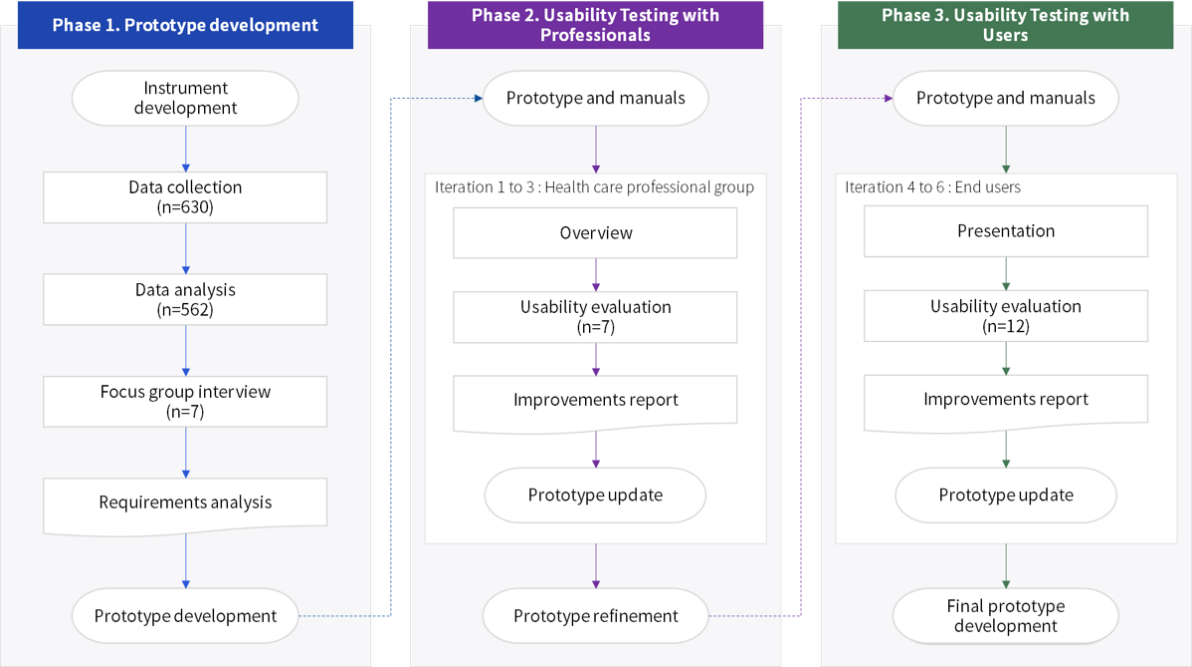
A scheme of the phases for a human-centered design approach to developing a worker-centered personal health record app.

This study applied the HCD methodology to design and develop a worker-centered PHR app (Figure 2). The goal was to develop a prototype based on quantitative and qualitative data analysis, and improve it through repeated usability evaluations. After defining the features of the prototype PHR app through benchmarking and a literature review, a questionnaire was developed through consultation with a group of health care professionals with advanced practice nurse licenses, including occupational health managers with experience in computerization in the workplace, a public institution practitioner, and a professor. The questionnaire consisted of 17 items, of which 12 enquired about participants’ general characteristics (sex, age, marital status, education, workplace, etc.), and 5 were configured to allow up to 3 responses on required data items and app features. Next, with the cooperation of the Korea Occupational Safety and Health Agency, we conducted a survey among workers in industrial parks in Korea, who had visited workers’ health centers (21 in total). Considering regional distribution, we included 30 workers from each center. We explained the background and purpose of the study, as well as the envisioned PHR app, to the participants, and questionnaires were distributed to those who had provided their consent. The survey was conducted for approximately 3 weeks (from November 9, 2018, to November 30, 2018). In total, 630 questionnaires were distributed and 575 were collected. Of the collected questionnaires, 13 were excluded because they did not meet study aims or included insincere responses, thus being inappropriate for analysis.

We conducted a frequency analysis of participants’ demographic data, and multiple response analysis of data items and app feature preferences. The results were relayed within FGI with the health care professional group, to inform the design of user profiles, requirements, interface concepts, and information architecture for the PHR app before developing the prototype.

### Usability Study

The prototype was evaluated by the health care professional group and end users (ie, workers who will be using the app). The health care professional group received applications from occupational health practitioners interested in participating in the study and usability evaluation. Participants in the evaluation study were selected based on their experience and occupation. The health care professional group meeting was conducted in a conference room with a large table to allow interaction among participants. First, we distributed the use cases and manuals to the health care professional group. Next, we performed a cognitive walkthrough of the prototype. For the health care professional group, we evaluated the usability of the scenario every time the task was completed with the After-Scenario Questionnaire (ASQ) [58], and the usability of the prototype was evaluated using the System Usability Scale (SUS) [59]. The SUS consisted of 10 items rated on a 5-point scale, ranging from 1 (Strongly disagree) to 5 (Strongly agree); it was converted into a total score between 0 and 100 points to evaluate the entire system. The ASQ consisted of 3 items, rated on a 7-point scale, ranging from 1 (Strongly disagree) to 7 (Strongly agree); each item of the ASQ evaluated the effectiveness, efficiency, and satisfaction of the task.

After reflecting on the prototype improvements derived through this process, the usability evaluation was performed for end users. The end users received online applications from individuals interested in participating in the study and usability evaluation, and the final participants were selected through random selection. End user evaluations were conducted individually to ensure privacy. The same methodology used for the health care professional group was applied to the 12 workers who participated in the usability evaluation; the research manager introduced the features of the app before performing the task, demonstrated the unique features of the app, and participants suggested improvements during interviews held after the task had been performed. The usability evaluation was conducted for approximately 6 months (from January to June 2019) and a total of 6 iterations were performed, 3 per group. At the end of each iteration, the prototype was improved based on the analyzed qualitative data, and tests were performed on existing participants (ie, 7 health care professionals and 12 end users). This study was conducted with the approval of the Korea Occupational Safety and Health Agency after a review of its research ethics (No. 211960314-00).

### Structural and Semantic Interoperability

Before designing a PHR with guaranteed interoperability, we analyzed data from various sources and structured workers’ PHR items by category. The basic information category comprised demographic information, personal history, family history, occupational history, and lifestyle. Data on these variables were collected by analyzing the database schema of the integrated system used in the workers’ health centers, and the document received from 5 occupational health managers.

The treatment and prescription history category consisted of hospital information, visit date, treatment type, hospitalization days, pharmacy information, medication frequency, and drug information. Data on these variables were collected by linking public data provided by the National Health Insurance Service and the Health Insurance Review and Assessment Service.

The medical checkup category referred to general medical checkup undertaken by the National Health Insurance Service, special medical checkup undertaken by the Korea Occupational Safety and Health Agency, target harmful factors, test methods, reference values, and units for each test item. Data on these variables were collected by analyzing the medical checkup results table and workers’ medical checkup guidelines.

The standardization process was performed after establishing content validity (selection of items, review of classification, reference value, and units, etc.) of the structured PHR items of workers; validity was evaluated by 5 occupational health managers. For structural interoperability, workers’ PHR was modeled through mapping between resource subitems and inspected PHR items after selecting FHIR resources corresponding to each category. For semantic interoperability, an appropriate code was defined through mapping between the concepts of SNOMED-CT and LOINC for the item representing the measured value of users. The mapped results were cross-validated by 2 experts: a laboratory medicine specialist and a medical informatics and nursing PhD graduate (HK).

### Architecture

**Figure 3:**
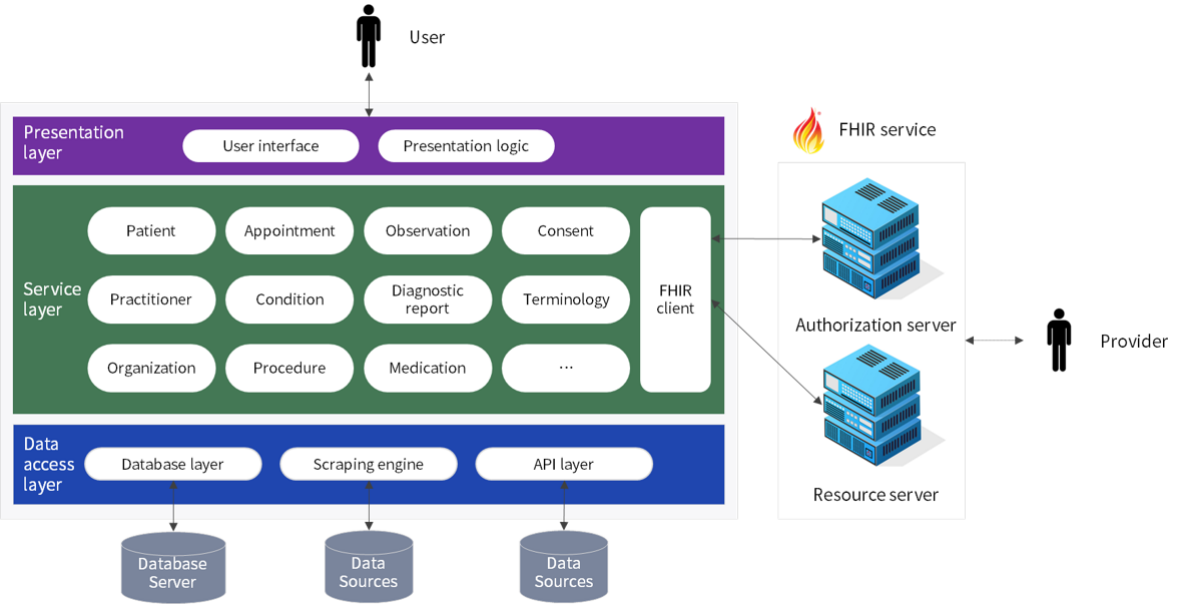
The architecture of the interconnected personal health record app Workcare using FHIR. API: application programming interface; FHIR: Fast Healthcare Interoperability Resources.

We developed a PHR app, named Workcare, which enables workers to systematically collect and store their health information from various sources and devices, and receive continuous health care services through data sharing. Workcare is an interconnected PHR app that secures ease of data entry, updates data using national health care data sets, guarantees the interoperability of PHR through a standardization process, and provides features for workers’ health management through the linkage between independent modules (Figure 3).

The data access layer collects users’ health information from various sources and stores it in a database. After user authentication, the API layer requests data through the API provided by each institution, and parses the response data; this allows access to test results and consultation records stored at the workers’ health center, or information from institutions utilized by the user, gathered from the hospital and pharmacy information provided by the Health Insurance Review and Assessment Service. The Scraping Engine is a screen scraping module developed to collect information from institutions that do not currently provide an API. This module first processes the authentication agent of a web service using a certificate stored in the smartphone, and then delivers the session to process the content of a specific site. In this way users can access multiple institutional websites to collect scattered health information by providing authentication information only once. The database layer stores users’ collected health information in a database, and updates data generated by the events of users and third parties. The data exchange between the client and the database server complies with the JavaScript Object Notation (JSON) through hypertext transfer protocol over secure socket layer (HTTPS). Data are securely transmitted by applying secure socket layer (SSL), and personal information is encrypted and decrypted by applying ARIA256 and SHA256.

The service layer implements the function of the PHR app through linkage between other layers. In this study, the functions were configured according to the HL7 PHR-S FM [60], a framework that lists the functions required or desirable for PHR, and complied with the standardized model of the PHR system. This procedure combines the FHIR resources (patient, appointment, observation, etc.) and FHIR client to provide the essential functions defined in the PHR-S FM. The FHIR client implements interconnected PHR services through the linkage between FHIR services. This converts health information that was collected based on the modeled workers’ PHR into FHIR resources, and then transmits the PHR to the FHIR service, or even parses the PHR delivered to the FHIR service. Notwithstanding, before data exchange with the resource server, user authentication and authorization are checked from the authentication server through OAuth 2.0, and only those authorized by the users can access their resources. We employed the Azure API cloud services, provided by Microsoft, for FHIR services [61].

The presentation layer provides users with the user interface/user experience for using the PHR app. Workcare was developed as a hybrid app, thus providing the same screen for the user, regardless of the resolution of the operating system (Android, iOS) or device type, thus complying with the mobile design guidelines derived from the improvements report extracted through the usability evaluation.

## Results

### Quantitative Data Analysis

Participants were 562 workers who visited 21 workers’ health centers in Korea. Most workers were women and older than 50 years, followed by those in their 40s and 30s. The most common duration of employment in the workplace was 1-4 years, and 63.9% (359/562) of the participants were employed in workplaces with less than 50 employees. Clerical and service-based businesses were more common than production and technical businesses (Table 1).

The results of the multiple response analysis for data items and feature preferences of the PHR apps are shown in Table 2. Regarding lifelogs to track, medication was the preferred feature, followed by the step count and diet. Regarding health data to track, blood pressure, weight, and blood glucose outranked body composition, body temperature, and oxygen saturation. Regarding information to manage, the highest preference was for examination result and the lowest for exercise. In terms of workplace health promotion, the preferences were, in order, for content provision, consultation record inquiry, and expert consultation. For other features, the preferences were, in order, data linkage, disease prediction, and material safety data sheet inquiry.

**Table 1:** Participants’ characteristics (N=562).

Characteristic		Value, n (%)
**Sex**		
	Male	195 (34.7)
	Female	367 (65.3)
**Age (years)**		
	<20	7 (1.2)
	20-29	94 (16.7)
	30-39	132 (23.5)
	40-49	155 (27.6)
	≥50	174 (31.0)
**Marital status**		
	Single	207 (36.8)
	Married	345 (61.4)
	Widowed	7 (1.2)
	Divorced or separated	3 (0.5)
**Education**		
	Middle school	21 (3.7)
	High school	142 (25.3)
	College (2 years)	87 (15.5)
	College (4 years)	270 (48.0)
	Graduate school	42 (7.5)
**Duration of employment in the workplace (years)**		
	<1	102 (18.1)
	1-4	227 (40.4)
	5-9	95 (16.9)
	≥10	138 (24.6)
**Number of employees in the workplace**		
	<5	62 (11.0)
	5-9	75 (13.3)
	10-29	98 (17.4)
	30-49	124 (22.1)
	50-99	37 (6.6)
	≥100	166 (29.5)
**Type of business**		
	Production	59 (10.5)
	Clerical	227 (40.4)
	Service based	185 (32.9)
	Technical	33 (5.9)
	Other	58 (10.3)
**Previous experience with health care app**		
	Yes	189 (33.6)
	No	373 (66.4)

**Table 2:** Summary of data items and feature preferences for the personal health record app.

Contents			Value, n (%)
**Lifelogs to track (n=1040)**			
	Medication	272 (26.15)	
	Step count	257 (24.71)	
	Diet	159 (15.29)	
	Stress	89 (8.56)	
	Exercise	86 (8.27)	
	Smoking	54 (5.19)	
	Drinking	48 (4.62)	
	Caffeine	45 (4.33)	
	Water	30 (2.88)	
**Health data to track (n=1024)**			
	Blood pressure	352 (34.38)	
	Weight	272 (26.56)	
	Blood glucose	249 (24.32)	
	Body composition	87 (8.50)	
	Temperature	38 (3.71)	
	Oxygen saturation	26 (2.54)	
**Information to manage (n=1196)**			
	Examination result	239 (19.98)	
	Health data	221 (18.48)	
	Prescription history	187 (15.64)	
	Lifelogs	182 (15.22)	
	Diet	143 (11.96)	
	Treatment history	142 (11.87)	
	Exercise	82 (6.86)	
**Workplace health promotion (n=1178)**			
	Content provision	314 (26.66)	
	Consultation record inquiry	285 (24.19)	
	Expert consultation	244 (20.71)	
	Reservations	152 (12.90)	
	Campaigns	101 (8.57)	
	Community	82 (6.96)	
**Other features (n=1224)**			
	Data linkage	289 (23.61)	
	Disease prediction	235 (19.20)	
	Material safety data sheet inquiry	234 (19.12)	
	Body age analysis	198 (16.18)	
	Health questionnaire	181 (14.79)	
	Medical institution inquiry	87 (7.11)	

### Qualitative Data Analysis

#### Overview

In total, 19 participants were part of the usability evaluation, including 7 health care professionals (Table 3) and 12 end users (Table 4). Most health care professionals were women, and most were in their 40s. They were licensed as advanced practice nurses. Their most common occupation was occupational health manager; all had more than 5 years’ experience in the related field, and 4 had previously used health care apps.

Similar to the health care professional group, most end users were women and in their 40s. Most had been employed in the same workplace for 5-9 years, and 5 had previously used health care apps.

The usability of the scenario (Table 5) and the prototype (Table 6) improved the results according to the iteration. The final ASQ was evaluated at a high level, with an average score of 5.90 (SD 0.43) out of 7. The final SUS was evaluated at an average score of 88.7 (SD 4.83) out of 100.

**Table 3:** Characteristics of health care professionals (N=7).

Characteristic		Value, n (%)
**Sex**		
	Male	1 (14)
	Female	6 (86)
**Age (years)**		
	30-39	2 (29)
	40-49	4 (57)
	≥50	1 (14)
**Marital status**		
	Single	1 (14)
	Married	6 (86)
**Education**		
	College (4 years)	1 (14)
	Graduate school	6 (86)
**Career(years)**		
	5-9	5 (71)
	≥10	2 (29)
**Type of occupation**		
	Occupational health manager	5 (71)
	Professor	1 (14)
	Official	1 (14)
**Previous experience with health care app**		
	Yes	4 (57)
	No	3 (43)

**Table 4:** Characteristics of end users (N=12).

Characteristic			Value, n (%)
**Sex**			
	Male	3 (25)	
	Female	9 (75)	
**Age (years)**			
	30-39	2 (17)	
	40-49	7 (58)	
	≥50	3 (25)	
**Marital status**			
	Single	3 (25)	
	Married	9 (75)	
**Education**			
	High school	4 (33)	
	College (2 years)	2 (17)	
	College (4 years)	6 (50)	
**Duration of employment in the workplace (years)**			
	1-4	2 (17)	
	5-9	7 (58)	
	≥10	3 (25)	
**Previous experience with health care app**			
	Yes	5 (42)	
	No	7 (58)	

**Table 5:** Usability evaluation results of scenario’s task^a^.

Section		Task 1^b^	Task 2^c^	Task 3^d^	Task 4^e^	Task 5^f^	Task 6^g^	Average	Target	
**Phase 2**									Health care professional group	
	Iteration 1	5.29 (0.41)	4.67 (0.33)	5.38 (0.49)	4.95 (0.33)	4.62 (0.33)	5.33 (0.33)	5.04 (0.37)		
	Iteration 2	5.67 (0.25)	5.24 (0.41)	5.86 (0.47)	5.00 (0.49)	5.10 (0.33)	5.67 (0.41)	5.42 (0.39)		
	Iteration 3	6.19 (0.49)	5.62 (0.49)	6.29 (0.41)	5.76 (0.56)	5.76 (0.47)	6.10 (0.31)	5.95 (0.46)		
**Phase 3**									End users	
	Iteration 4	5.22 (0.34)	4.53 (0.29)	5.28 (0.38)	4.56 (0.42)	4.61 (0.48)	5.31 (0.48)	4.92 (0.40)		
	Iteration 5	5.64 (0.32)	5.17 (0.34)	5.78 (0.61)	5.03 (0.46)	5.17 (0.47)	5.56 (0.56)	5.39 (0.46)		
	Iteration 6	6.17 (0.43)	5.53 (0.24)	6.22 (0.37)	5.61 (0.42)	5.69 (0.32)	6.17 (0.59)	5.90 (0.43)		

^a^All values are presented as mean (SD).

^b^Task 1: After entering your account information, log in to the personal health record app.

^c^Task 2: After providing certification, import the national health care data sets.

^d^Task 3: After adding health data values, look at the stored values.

^e^Task 4: Select the data sharing range and upload the personal health record to the Fast Healthcare Interoperability Resources service.

^f^Task 5: After connecting the system, use the linked institutions’ services.

^g^Task 6: Use the services after checking the provided occupational health content.

**Table 6:** Usability evaluation results of prototype.

Section		Mean (SD)	Target
**Phase 2**			Health care professional group
	Iteration 1	83.2 (3.45)	
	Iteration 2	85.4 (2.50)	
	Iteration 3	86.9 (1.73)	
**Phase 3**			End users
	Iteration 4	86.2 (6.83)	
	Iteration 5	87.2 (5.05)	
	Iteration 6	88.7 (4.83)	

#### Suggested Improvements: Health Care Professionals

The major improvements derived from the usability evaluation of the health care professional group are provided below. The health care professional group suggested improvements for the features and contents of the PHR app, and improvements with similar contents were integrated into a single category.

##### Lifelogs

Medication, smoking, and alcohol are essential items for managing workers' lifestyle habits and calculating risk factors for cerebrovascular disease. If workers can calculate risk factors for developing cerebrovascular diseases by data collected via the PHR app and self-tests, it will be a motivation for health management.

Health care professional 6

Since the type of food, calories, and nutritional contents differ by database, accurate information [about food intake] cannot be recorded and managed [in the app]. Also, according to past experiences of using existing health care apps, the process of searching and recording food intake was cumbersome.

Health care professional 1

##### Medical Checkup

Most construction workers are daily workers, so it is not easy to manage their medical checkup results. If daily workers can manage their individual medical checkup results through the PHR app, it will be of great help to occupational health managers who have recently moved to new workplaces.

Health care professional 2

Medical checkup results are sent to individual workers, and workers often lose them, so they do not bring them when consulting with an occupational health manager. The PHR app should enable the easy sharing of PHR to occupational health managers through user authentication and consent.

Health care professional 1

Most older adult workers do not have a certificate on their smartphones. In consideration of these classes, it is necessary to improve the feature of the app, so that the medical checkup results can be managed as images.

Health care professional 3

##### Harmful Factors

Even if workers are trained through material safety data sheets, they must be notified by the occupationalhealth manager. If it is possible to provide information on harmful factors for each user's work area through the PHR app, it may greatly help occupationalhealth managers' work convenience and workers' access to information.

Health care professional 5

It would be helpful if we could provide customized content according to the user's business and occupation. For example, it would be of great significance if workers could check information on precautions and harmful factors for their assigned processes through the PHR app.

Health care professional 7

#### Suggested Improvements: End Users

The major improvements derived from the usability evaluation of the end users are provided below. Generally, end users suggested improvements for data handling, and improvements with similar contents were integrated into 1 single category.

##### Data Input

I wish there were various ways to enter the result values. If I have to enter each value through the keypad, I think this will be a barrier for me to perform data entry.

End user 10

I would like to add a feature that can record the location in which I conducted the measurement. In my case, I tend to measure and record blood pressure and blood glucose in various places, such as my home, workplace, and the hospital.

End user 2

##### Data Output

It was difficult to concomitantly check the trends and values when there were separate lists and graphs, like in other existing health care apps. I wish I could see graphs and lists together on one screen.

End user 3

It should be possible to compare, at a glance, my current results with past medical checkup results. If you need to separately check the results of the medical checkup for every year, like now, it becomes inconvenient to check the trend of items that I want to carefully examine. Also, I wish I could see the categories of values of specific measurements according to a reference value.

End user 8

It was good to be able to check the dosage guide and precautions [about a drug] in the prescription history. Can you not add the image of the drug?

End user 4

##### Data Sharing

Can I not select the range (item and date, etc.) of information that I wish to share? I agree to share data for continuous health care services, but there are specific data that I do not want to share.

End user 1

##### Data Security

Do you have any plans to add security features to the app? Even if the smartphone has a lock feature, it seems that a second authentication feature (fingerprint and password, etc.) is required to protect the sensitive personal information in the PHR app.

End user 2

### PHR Modeling

**Figure 4:**
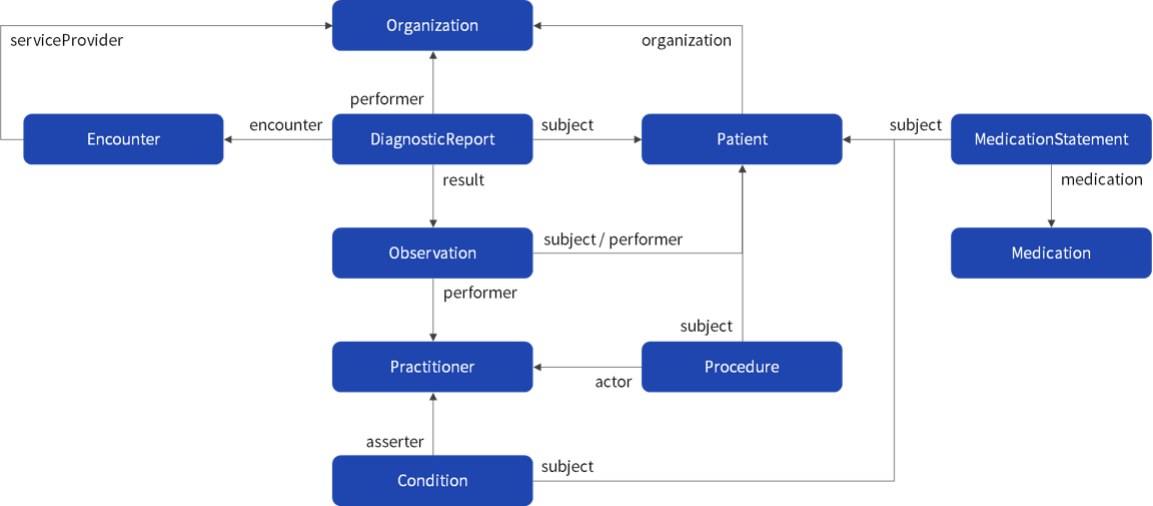
FHIR resource diagram of workers’ personal health record.

Among the FHIR resources, the structured PHRs of workers are shown in Figure 4. The Patient resource could be used to relay all information about patients and their surroundings, although this study focused only on representing workers’ personal information. The Organization resource represented information from not only the workplace but also all other organizations used by workers, such as hospitals, pharmacies, and examination centers, collected through health care data sets. The DiagnosticReport resource could be used to describe a doctor’s opinion based on information about a specific medical service and data measured in that medical service, although this study focused only on describing types of medical checkup and a doctor’s opinion about the checkup. MedicationStatement and Medication resources were used for describing the prescription history, and the Procedure resources for relaying medical history and consultation records. Workers’ PHR based on these resources were included in the Bundle resource and processed as a set when FHIR services interacted.

Among the workers’ PHR items, items requiring mapping comprised 40 general medical checkups, 289 special medical checkups, and 18 lifelogs (Table 7). General medical checkups are conducted for the early detection/prevention of diseases in workers, their dependents, and local subscribers. The types of examination for general medical checkups comprised general medical tests, oral tests, position tests (eg, for height, weight, obesity, and blood pressure), chest radiation, urinalysis, and blood examinations, etc., and the examination items differed by sex and age of the worker.

Special medical checkups are conducted to prevent occupational diseases and manage the health of workers engaged in jobs that expose them to harmful factors. Because there is a standardized test for each of the 179 harmful factors regulated by Korea’s Occupational Safety and Health Act (eg, *N*, *N*-dimethylacetamide, benzene, acrylonitrile, vinyl chloride, dust), the test items for special medical checkups differed by work environment of the workers.

The lifelogs comprised items generated in daily life (eg, the number of steps and exercise) and about lifestyle (eg, the amount of drinking and smoking). As a result of the mapping, 347 items, except for 41, were mapped with the concepts of SNOMED-CT and LOINC.

**Table 7:** Mapping results of workers’ personal health record items.

Section	Count	Mapping		Nonmapping
		SNOMED-CT^a^	LOINC^b^	
General medical checkups	40	40	35	—
Special medical checkups	289	208	234	41
Lifelogs	18	18	11	—
Total	347	266	280	41

^a^SNOMED-CT: Systematized Nomenclature of Medicine–Clinical Terms.

^b^LOINC: Logical Observation Identifiers Names and Codes.

Most items that served as diagnostic tests (eg, general and special medical checkups) could be mapped with the concept of LOINC, although those that did not serve as diagnostic tests could not be mapped. Items that needed to be described in words rather than numbers (ie, doctor’s opinion, medical history, occupational history) were mapped with the concept of SNOMED-CT and expressed as precoordinated, and items that needed to be partially specified were expressed as postcoordinated. Nonmapping items required specificity because they were ambiguous. For instance, the leukocyte percentage item, which was included in the hematopoietic classification, exists in various LOINC concepts (770-8, 35332-6 19023-1, 736-9, 42250-1, 5905-5, 713-8, 706-2) depending on the type of leukocyte. To summarize, when generating FHIR-based PHR, the concept of SNOMED-CT was used for lifelogs items, and the LOINC as a priority for general and special medical checkup items.

### Final Developed Prototype App

Workcare provides users with PHR management according to the collection of data on workers’ lifelogs, vital signs, medical checkup results, health care services (eg, reservation and teleconsultation), occupational safety and health information (eg, material safety data sheet search), and a health questionnaire. Users can access these features through more than 200 screens, and the app has an intuitive navigation system that minimizes the number of actions that users need to perform for accessing the desired content. The configuration of the screen was made in a way that frequently used features (eg, dashboards, profiles, and specific content) are placed on the bottom tab, with each screen being placed on its appropriate tab according to feature type.

**Figure 5:**
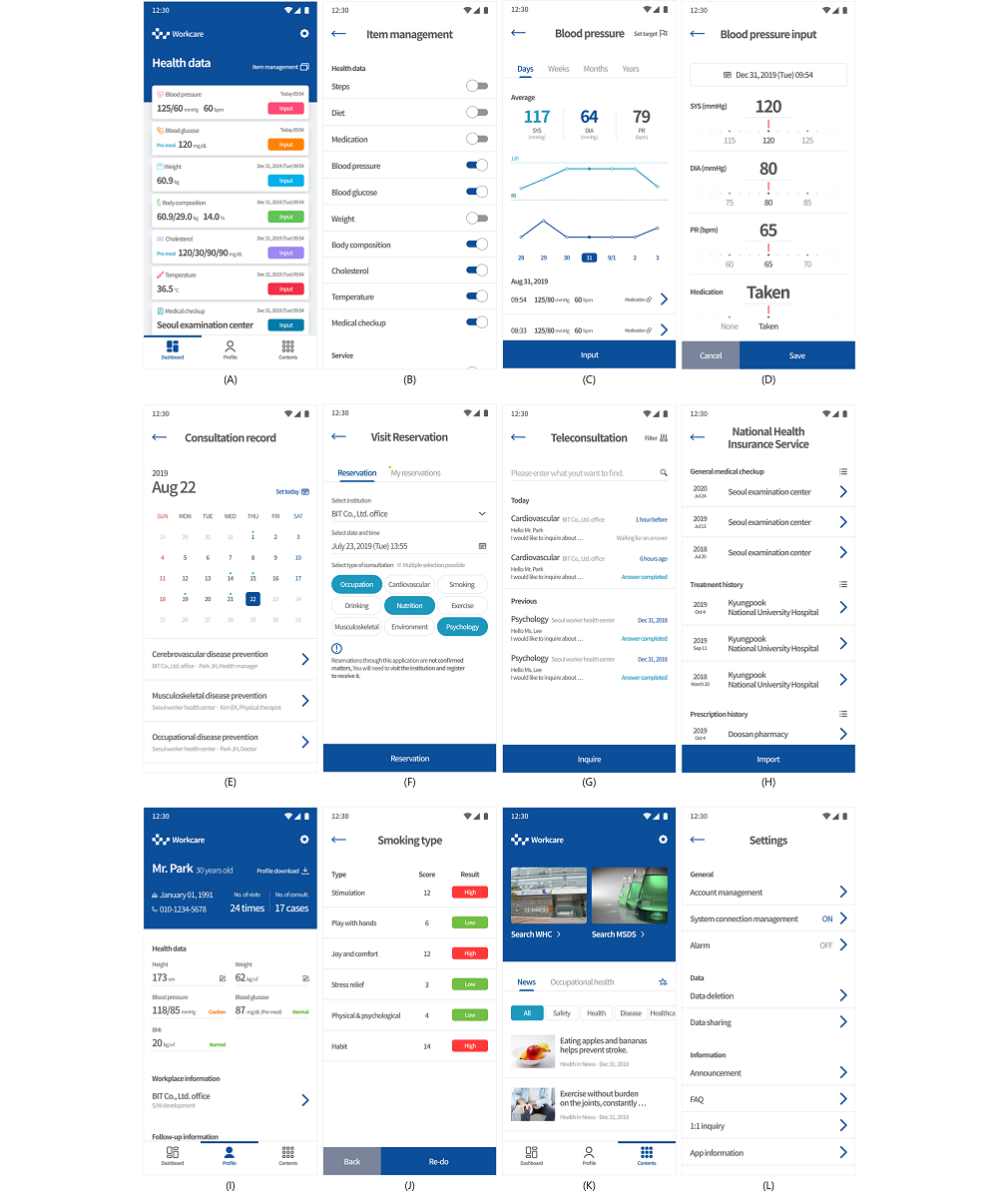
Screenshots of different functions in the worker-centered personal health record app.

The Dashboard tab (Figure 5A) provides the user with the main features for PHR management and health care services. The row in the Dashboard tab outputs the status of each feature, and frequently used features can be moved to the corresponding screen by clicking a button. For instance, the blood pressure row describes the latest measured value, and date and time at which it was measured; the user can also click the blood pressure row to move to the blood pressure screen (Figure 5C), or even click the input button to go to the blood pressure input screen (Figure 5D). Users can also enter the management screens for the number of steps, diet, medication, blood glucose, weight, body composition, cholesterol, body temperature, and general medical checkup results, or even view, through the API, values that were collected from and measured in various institutions. Users can also manage health-related tasks provided by API-linked institutions, such as consultation record (Figure 5E), visit reservation (Figure 5F), teleconsultation (Figure 5G), and data of the National Health Insurance Service (Figure 5H). The row within the Dashboard tab (Figure 5A) can select the order of items and the decision on whether to display them on the item management screen (Figure 5B) can be made by clicking the item management label at the top right.

Regarding health data (eg, blood pressure and glucose), the screens were configured in a pattern similar to that of the dashboard. Typically, the blood pressure screen (Figure 5C) allows users to check blood pressure information (average, graph, and list) according to day, week, month, and year through the upper tab; in this screen, users can manipulate the graph by swiping left and right, with the measured value in the lower list and the average value at the top being updated according to the selected x-axis (ie, day, week, month, and year) in the graph. From the blood pressure input screen, users can directly input blood pressure data (Figure 5D) by clicking the input button, or even automatically enter measured values from a blood pressure device that has been paired with the app. The consultation record screen (Figure 5E) allows users to check health consultation records for visits to various health institutions (eg, the workers’ health center). The visit reservation screen (Figure 5F) allows users to reserve a consultation in a specific institution; in the Reservation tab, users select the institution they wish to visit, and the date, time, and type of the consultation. In the My Reservations tab, users can check information about the reservation, cancel it, or call the institution that made the reservation. The teleconsultation screen (Figure 5G) allows users to check responses from institutions after making an inquiry about health consultations; after reviewing the PHR shared through the FHIR service with medical personnel in the institution, users can also check the message sent to an institution. After completing certification in the login screen, users can collect and check the health care data present in public institutions; in other words, after completing certification of security and logging in the login screen, the National Health Insurance Service screen (Figure 5H) becomes available to users, who can then save their medical treatment history, prescription history, medical checkup results, and medical institution information by clicking the import button. Saved data can be viewed in detail on the screen by clicking a row.

The Profile tab allows users to check the main information of the user who is logged in (Figure 5I). In the upper area, basic information (eg, users’ name, date of birth, and phone number) is described, with the health information of the user being output below the basic information in the upper area. By clicking on the Blood pressure, Blood glucose, and Body mass labels, users can check the share of the measured values according to a reference value through a graph that appears on the screen. By clicking on the workplace information row, users are moved to the screen that outputs information related to the workplace to which users belong; by clicking on the follow-up information row, users are moved to a screen that outputs the doctor’s opinion about the results of the medical checkup. Based on the collected data and on the health questionnaire, users can self-evaluate their risk of cerebral heart disease, risk factors of cerebral cardiovascular disease, cerebral cardiovascular disease occurrence probability, and body age. Users can undergo health questionnaires on the smoking type (Figure 5J), nicotine dependence, job stress, psychological stress, and check the trend of the results.

The Contents tab (Figure 5K) provides users with information on occupational safety and health. In the upper area, images are arranged in a way to allow users to search for workers’ health centers and material safety data sheets. The lower area outputs a list containing useful news and information on occupational safety and health. By clicking on the workers’ health center search, users can check the locations, phone numbers, and home pages of 23 workers’ health centers nationwide. The material safety data sheet provides detailed information on 16 categories, including chemical hazards, first aid measures, countermeasures in case of chemical exposure, and toxicity information; users can click a star icon to select a topic they wish to be displayed in the favorites screen.

The settings screen (Figure 5L) provides users with the main features for configuring the app environment. The account management row allows users to select whether they want to automatically log-in, change password, log out, or cancel their membership. The system connection management row displays a list of systems that have requested access to users’ resources through the FHIR service, and users can add or delete these connections. The alarm row allows users to configure the app to produce push messages for major events, such as reservations, health counseling appointments, and goal achievements. The data deletion option allows users to delete all their data (after self-certification), while the data sharing option allows for uploading and synchronizing users’ PHR according to the selected item and date. Finally, users can check important information necessary for service use through announcements, frequently asked questions, 1:1 inquiry, and app information.

## Discussion

This study aimed to develop a PHR app that can provide worker-centered interconnected PHR services to support workplace health promotion by using health care standards, cloud services, and national health care data sets to solve known major challenges of PHR (ie, interoperability, security and privacy, and data quality), and by applying the HCD methodology to design an app based on users’ perspectives.

We designed a service that integrates workers’ health information that is scattered across various sources, and manages PHR through FHIR services; we used national health care data sets to ensure data entry, update, and quality. In 2017, the Republic of Korea revised the Act on Providing and Utilizing Public Data to guarantee the public’s right to know about and access public data, as well as to ensure that most institutions provide data sets to the public. Accordingly, the National Health Insurance Service, while operating the national health insurance system, built a database comprising information on medical treatment history, prescription history, medical checkup results, and medical institution information; this database allows Korean citizens to check their data through self-certification. To prevent occupational diseases in workers, medical personnel need data on patients’ treatment and prescription history, medical checkup results, and workers’ PHR, as such thorough data can support medical personnel’s decision making. Knowing the inherent problems of PHR (ie, regarding data input, update, and quality), we endeavored to acquire high-quality data that are managed by the Korean government through an interface method with institutions related to the management of workers’ PHR. Nonetheless, the type of data that are measured by workers’ visits to health institutions (eg, medical checkup results) has limitations regarding the identification of workers’ health status at specific periods. Therefore, the PHR app we developed allows workers to measure and store PGHD through various devices, as well as to include these data in workers’ PHR, so that medical personnel can identify workers’ status even during periods when they will not or cannot visit a health institution.

Interconnected PHR is the ideal implementation of PHR, but the literature reports hindrances in standardizing the format and terminology for PHR information exchange. Previous studies on PHR have been conducted, but they differ from our study in several ways. First, previous studies [34,37] using document standards (eg, CCR and CDA) treated PHR as a single document; therefore, in previous studies the entire document must be updated when updating a single item. By contrast, PHR using FHIR, such as the one we used, does not incur such problems; items can be updated separately because they are managed in a server by resource unit. Second, previous studies [35,37] that developed tethered PHR are dependent on specific electronic medical records (EMRs) and EHR; our app is not dependent on a specific system because the information is collected from various sources and is integrated and managed in the FHIR service according to the users’ will. Besides, the users can have complete management authority over their health information. Third, some previous studies [38,39] used FHIR for managing PHR, but did not address privacy, security, and authentication issues. Our study, notwithstanding, developed an app that requested user authentication, confirmation, and authorization to access health resources through OAuth 2.0, also applying SSL, ARIA256, and SHA256 to solve privacy and security issues.

To ensure user convenience and usability, we designed the PHR app while considering the users’ perspective through the HCD methodology. Previous studies [30,62-65] have shown that users expect PHR apps to assist in their health management by providing user-friendly and patient-centered features. Hence, this study considered data items, features, and interfaces that are suitable for user profiles through both quantitative and qualitative data analysis. Data items comprised lifelogs (eg, number of steps, diet, medication), health data (eg, blood pressure, blood glucose, and weight), medical checkup data (ie, general and special medical checkup results), and treatment and prescription history data. According to a systematic review of the literature by Roehrs et al [30], the common data items in PHR were allergy, vaccination, test results, and drugs, with little data on vital signs. Originally, we included allergy and vaccination items in the questionnaire of this study, but they were excluded through consultation with a health care professional group; this exclusion occurred because these items were considered less important than other items for occupational health.

Accordingly, we were able to derive various improvements to the app by conducting usability evaluations with both a health care professional group and an end user group. Regarding older adult workers, we added a feature to manage and show medical checkup results as an image; based on the opinion of the health care professional group, 1 out of 3 end users aged over 50 years are likely to not have a certificate on their smartphone, and thus, they would not be able to save the medical checkup results. Thus, amid the improvements to our prototype, we developed a feature that allowed users to directly input medical checkup results, capture a picture of the results through a smartphone camera, and save the picture. Moreover, we improved the diet management feature of the app using the integrated database; albeit the end users confirmed the need for information on dietary preferences through the survey results, the health care professional group did not confirm this addition because they were concerned about the lack of a unified system for the type of food, calories, and nutritional contents. We used the food nutrition ingredient database provided by the Ministry of Food and Drug Safety to solve the concerns of the health care professional group. The inconvenience of data recording about food, remarked by the health care professional group as another concern, was also dealt with by developing a feature to include frequently searched food (My Food) and image add-ons.

We developed a PHR app that can support workers’ self-health management. Through the app, workers can collect and monitor their health information through the Dashboard tab, schedule a visit to a linked institution, or receive teleconsultations. The data items of this study were similar to those of a previous study [46], but some items (eg, water, alcohol, and smoking) were not included in the app. These items showed a low preference in the survey results of our study, and through the usability evaluation interview, we confirmed that users deemed the recording of frequent daily behaviors (eg, water and alcohol intake and smoking) as difficult. Therefore, we saved data on users’ lifestyles through the inclusion of a health questionnaire for evaluating the risk of cerebrovascular disease, not through specific data collection items. The Profile tab allows users to check the status of their measured value according to a reference value, or even to check their cerebrovascular disease evaluation and body age based on the collected data. Zhou et al [42] did not support the analysis of data input by users; thus, this feature may have a limitation, in that the status of the measured values cannot be checked. In this study, we used the reference value of the structured workers’ PHR item to determine whether the measured value of each item is normal. Nevertheless, based on the guideline [66] of the Korea Occupational Safety and Health Agency, it is possible to probabilistically predict the development of cerebrovascular disease by analyzing users’ stored data on lifestyle and medical checkup results.

Recent changes in the social environment caused by COVID-19 have had a great impact on the distribution and production industries. The explosive increase in the volume and sorting of parcel deliveries owing to the COVID-19 pandemic led to the overwork of parcel workers, triggering an opportunity for the government and companies to review the state of workers’ health improvement in the workplace. However, after the establishment of the employee assistance program provided by the Ministry of Employment and Labor in 2007, Korean workers have been provided with limited offline consultation opportunities; most employee assistance programs in Korea focus on mental health care, while research and investment in workers’ health care services using technology have been insufficient. This study was, to the best of our knowledge, the first to develop a PHR app suitable for occupational health in Korea. Our PHR app can contribute to workers’ personal health management by improving accessibility to their data and enabling the collection and management of their health information held by various institutions in one place. Registered users can continue to receive occupational health services by accessing and viewing their PHR at other institutions that comply with standards, even if they leave the workplace. This lays the foundation for ultimate workplace health promotion.

Most previous studies have focused on developing a PHR app for patients and older adults, while few researchers have endeavored to develop a PHR app for workers and support workplace health promotion. Thus, this study is meaningful in that it developed a worker-centered PHR app for workplace health promotion; however, it also had limitations. We attempted to integrate workers’ health information that was scattered across various sources, but did not include data from hospitals. In order to activate worker-centered data exchange, hospital participation is essential, and data standard issues must be resolved for each hospital. Even though Korea’s EMR introduction rate is over 90%, it is difficult to utilize these data due to low standardization levels. The PHR app we developed enables information exchange between systems that comply with the standard through the FHIR service; however, a medical infrastructure that can guarantee continuity of treatment to patients is currently being developed in Korea. Since 2018, the national project for enabling the exchange of medical information between hospitals has been expanded with an EMR certification system (a system to verify national standards and conformity for EMR has been implemented in June 2020). Despite these advances, the possibility of integrating data from EMRs of hospitals visited by workers was still limited at the time of this study. However, given that EMRs include relevant health-related data (eg, vital signs, drugs, allergies, test results, and radiographic images), we believe that linkage between EMRs is necessary to ensure the provision of a wider number of services for users. Future studies are warranted to confirm the exchange of workers’ medical information through the linkage between systems that have received the EMR certification system in Korea, and design a PHR app for workers that includes EMR data. Accordingly, future research may expand the service range of Workcare by linking it with the cloud EMR of BIT Computer Co. Ltd., to which the lead author (HP) is affiliated. Further, to confirm the clinical effectiveness of PHR services in the workplace, case–control and prospective studies will be conducted, and studies to analyze the satisfaction of workers and medical personnel with PHR services will also be conducted.

## Abbreviations

API: application programming interface
ASQ: After-Scenario Questionnaire
CCR: continuity of care record
CDA: clinical document architecture
DICOM: Digital Imaging and Communications in Medicine
EHR: electronic health record
EMR: electronic medical records
FGI: focus group interview
FHIR: Fast Healthcare Interoperability Resources
HAPI: HL7 Application Programming Interface
HCD: human-centered design
HL7: Health Level Seven
HTTPS: hypertext transfer protocol over secure socket layer
JSON: JavaScript Object Notation
LOINC: logical observation identifiers names and codes
PGHD: patient-generated health data
PHR: personal health record
SNOMED-CT: Systematized Nomenclature of Medicine–Clinical Terms
SSL: secure socket layer
SUS: System Usability Scale
UCD: user-centered design
WHO: World Health Organization
